# Power frequency magnetic field promotes a more malignant phenotype in neuroblastoma cells via redox-related mechanisms

**DOI:** 10.1038/s41598-017-11869-8

**Published:** 2017-09-13

**Authors:** S. Falone, S. Santini, V. Cordone, P. Cesare, A. Bonfigli, M. Grannonico, G. Di Emidio, C. Tatone, M. Cacchio, F. Amicarelli

**Affiliations:** 1 Department of Life, Health and Environmental Sciences, University of L’Aquila, L’Aquila, Italy; 2 Department of Neurosciences, Imaging and Clinical Sciences, University “G. d’Annunzio”, Chieti Scalo (CH), Italy; 3 Institute of Translational Pharmacology (IFT) - CNR, L’Aquila, Italy

## Abstract

In accordance with the classification of the International Agency for Research on Cancer, extremely low frequency magnetic fields (ELF-MF) are suspected to promote malignant progression by providing survival advantage to cancer cells through the activation of critical cytoprotective pathways. Among these, the major antioxidative and detoxification defence systems might be targeted by ELF-MF by conferring cells significant resistance against clinically-relevant cytotoxic agents. We investigated whether the hyperproliferation that is induced in SH-SY5Y human neuroblastoma cells by a 50 Hz, 1 mT ELF magnetic field was supported by improved defence towards reactive oxygen species (ROS) and xenobiotics, as well as by reduced vulnerability against both H_2_O_2_ and anti-tumor ROS-generating drug doxorubicin. ELF-MF induced a proliferative and survival advantage by activating key redox-responsive antioxidative and detoxification cytoprotective pathways that are associated with a more aggressive behavior of neuroblastoma cells. This was coupled with the upregulation of the major sirtuins, as well as with increased signaling activity of the erythroid 2-related nuclear transcription factor 2 (NRF2). Interestingly, we also showed that the exposure to 50 Hz MF as low as 100 µT may still be able to alter behavior and responses of cancer cells to clinically-relevant drugs.

## Introduction

The use of electric devices and equipments in clinical practice, industrial environments, and common domestic situations generate extremely low frequency magnetic fields (ELF-MF) with frequencies of 0–60 Hz, and magnetic flux densities up to 10 mT^1^. In 2002, the World Health Organization’s International Agency for Research on Cancer (IARC) classified ELF-MF as possible carcinogens for humans^2^. Like many other non-ionizing radiations, ELF-MF do not have enough energy to directly damage DNA, however they are suspected to play an important role in co-carcinogenesis, as well as in the progression of tumorigenesis^3–6^. Later stages of malignancies are linked to both metabolic rewiring and enhanced detoxification capacity, which are believed to provide critical proliferative or survival advantage^7–9^.

Some of us have previously shown that the exposure to an ELF magnetic field triggers a strong proliferative response in SH-SY5Y human neuroblastoma cells, and this is linked to the expression of novel proteins associated with a more malignant phenotype^10^. Since some ELF fields favor malignant cells proliferation, some authors have suggested that particular precaution is required for the use of ELF-MF-generating devices on cancer patients in medical, residential or industrial environments^6, 11^. More recently, we have shown that a power frequency (50 Hz, 1 mT) ELF magnetic field de-differentiates further SH-SY5Y cells, and switches their metabolism to the highly efficient mitochondrial respiration, which better meets the energy demands of rapid cell growth and frequent divisions^12^. Mitochondria represent a major production site for reactive oxygen species (ROS)^13^, against which malignant cells are well protected through the overexpression of crucial antioxidant enzymes, and this seems to be linked to tumor survival, progression and multidrug resistance (MDR)^14, 15^. Accordingly, the efficacy of many chemotherapies relies on the ability to overwhelm the ROS-scavenging capacity of tumors and cancers^15–18^. Some of us have also reported that human neuroblastoma cells respond to an ELF field by increasing the availability of reduced glutathione (GSH), a powerful endogenous thiol-based free radical scavenger^12^, thus confirming the shared opinion that the interaction between ELF-MF and biosystems may involve the perturbation of the cellular redox balance^19–26^. Besides being a critical mediator of chemoresistance in both neuroblastomas and gliomas^27, 28^, GSH is an essential co-factor for both antioxidant glutathione peroxidase (GPX) and phase II drug-metabolizing glutathione S-transferase (GST) enzymes, with the latter being one of the major determinants of MDR phenotype in tumor cells^29–31^. Among the major controllers of the cellular redox environment, sirtuins 1 and 3 (SIRT1 and 3), along with the master regulator erythroid 2-related nuclear transcription factor 2 (NRF2), have been recognized to play crucial roles in the cytoprotective response against oxidative challenge as well as in the onset of drug resistance phenotype, mainly through the transcriptional activation of key antioxidant and detoxifying enzymes, such as GPX, GST, superoxide dismutases (SOD) and catalase (CAT)^32–37^. In coherence with their strategic role in cellular protection, both SIRT1 and 3 are frequently over-expressed in several type of cancers, and contribute to chemo- and radio-resistance^38–41^. In addition, some authors have drawn attention to the constitutive activation of NRF2 in cancer progression and resistance to therapy^42–44^. Interestingly, it has been recently hypothesized that the exposure to an ELF-MF may alter the expression profile of both SIRT1 and NRF2^45, 46^, thus perturbing the systems that control the antioxidant cellular responses.

The possible link between environmental ROS-generating agents and the major redox-responsive defensive systems which are critically linked to tumor progression is fueling an ever-growing research activity that is aimed at clarifying how tumor cells are able to adapt critical cytoprotective networks in the presence of ELF fields, and whether this might trigger relevant changes in tumor phenotype or even accelerate the progression of cancers. Unfortunately, the reports available so far are mostly limited to short-term studies, and this may represent a major weakness as ELF electromagnetic fields are nearly omnipresent, thus making chronic exposure to multiple ELF fields an everyday experience for most people^47^.

On this basis, the aim of this study was to provide a proof-of-concept that the cytoproliferative response of human neuroblastoma cells under continuous exposure to a 50 Hz, 1 mT magnetic field could be associated with simultaneous cytoprotective adaptations involving the enhancement of key ROS-targeting and detoxification enzymatic systems, through the possible regulation of the upstream SIRT/NRF2-dependent control. In addition, since an altered tolerance of cancer cells against exogenous ROS-generating compounds may reflect changes in drug-inactivating capacity, and possibly the development of chemoresistance^48^, we also studied whether the vulnerability of neuroblastoma cells towards hydrogen peroxide and ROS-generating anti-tumor drug doxorubicin (DOXO) could be affected by the ELF-MF exposure. Finally, as it is suspected that ELF-MF lower than 1 mT (i.e., the new reference level established in 2010 by the International Commission on Non-Ionizing Radiation Protection guidelines for occupational exposures to power frequency ELF magnetic fields^49^) may result in significant alterations of cellular behavior^50^, we also wanted to verify whether the major effects that were elicited by 1 mT MF were also detected when neuroblastoma cells were exposed to a 50 Hz, 100 µT MF.

## Methods

### Antibodies

Abcam (Cambridge, UK) provided the following primary antibodies: anti-sirtuin1 (cat. ab12193; dil. 1:750), anti-sirtuin3 (cat. ab86671; dil. 1:1,000), anti-nuclear factor erythroid 2-related factor 2 (NRF2) phospho S40 (cat. ab76026; dil. 1:5,000 and 1:250, for Western immunoblotting and immunofluorescence, respectively), and anti-β-actin (cat. ab8227; dil. 1:10,000). The HRP Goat Anti-Rabbit IgG secondary antibody (cat. PI1000; dil. 1:1,000) was purchased from Vector Laboratories, Inc. (Burlingame, CA, USA). Thermo Fisher Scientific Inc. (Waltham, MA, USA) provided the AlexaFluorTM 488 goat anti-rabbit IgG (H+L) (cat. A-11008; dil. 1:1,000).

### Cell cultures

SH-SY5Y human neuroblastoma cells (cat. 94030304; Sigma-Aldrich, Milan, Italy) were seeded (1 × 10^4^ cells/cm^2^) and cultured as monolayer in moist air, at 37 °C, 5% CO_2_, in a cell incubator (model MCO-15AC; SANYO Electric Co., Ltd., Moriguchi, Japan), using 10% (v/v) foetal bovine serum (FBS)-supplemented RPMI-1640 medium (cat. ECS0180L and ECM0505L, respectively; Euroclone, Milan, Italy), containing 2 mM L-glutamine, 100 IU/ml penicillin and 100 mg/ml streptomycin (cat.10378-016; Life Technologies Italia, Monza, Italy), as described in previous papers^10, 12^.

### Exposure to the extremely low frequency electromagnetic field

Both generation and characterization of the magnetic field used in this study were performed by using instruments from RFL Industries (Boston, NJ, USA). In particular, solenoids produced a highly homogeneous electromagnetic field at 50 Hz frequency, with either 1 mT (10 Gauss) or 0.1 mT (1 Gauss) flux density, as previously described^10, 12, 20, 22^. In order to measure continuously the flowing current in the circuit, an AC meter was connected serially to the solenoid. Both waveform and frequency of the current, along with the magnetic field intensity, were monitored by using an oscilloscope and an axial Hall-effect probe 912 Digital Gaussmeter. No magnetic field-induced heating was observed. Cells were exposed to either sham or MF condition, choosing randomly which solenoid had to be powered. In order to keep the treated groups unknown to the researchers involved in the sample processing, code labels were used, and codes were undisclosed only at the end of data analysis. Any seasonal effect of the local geomagnetic static field (<50 μT) was avoided by processing control and MF-exposed cells simultaneously.

### Viable cell counting

Viable cell counting was performed by using a hemocytometer chamber after detachment of the monolayer with Trypsin/EDTA and Trypan blue 0.4% staining (cat. 25300-054 and 15250-061, respectively; Life Technologies Italia). Four independent experiments were carried out.

### Cell extract preparation for enzymatic activity assessments

Control and ELF-MF-exposed cells were harvested and lysed (5 × 10^7^ cells/ml) in: (a) 100 mM phosphate buffer (pH 7), containing 1.5 mM dithiotreitol (DTT) and 1 mM EDTA (for both total glutathione peroxidase and glutathione S-transferase); (b) 100 mM phosphate buffer (pH 7), containing 0.1% (v/v) Triton X-100 (for both total superoxide dismutase and catalase). Cell suspensions were homogenized and centrifuged at 14,000 × g for 30 min at 4 °C. The resulting supernatants were used for spectrophotometric measurement of total protein concentration (cat. 500-0006; Bio-Rad Laboratories, Milan, Italy), using BSA as the standard^51^, and for the assessment of all the enzymatic activities. All spectrophotometric readings were carried out in triplicate by using a Lambda25 spectrophotometer (PerkinElmer, Inc., Waltham, MA, USA).

### Measurement of total superoxide dismutase (tSOD) activity

Total SOD (EC 1.15.1.1) activity in cell extracts was assayed in 50 mM NaHCO_3_ buffer (pH 10.2), containing 25 mM EDTA and 0.1 M epinephrine bitartrate (cat. E4375; Sigma-Aldrich). SOD ability to inhibit the epinephrine auto-oxidation was monitored at 480 nm and 30 °C, according to Sun and Zigman^52^. One unit was defined as the amount of enzyme required to halve the rate of epinephrine autoxidation. Five independent experiments were carried out.

### Measurement of catalase (CAT) activity

The CAT (EC 1.11.1.6) activity was assayed by recording at 240 nm and 25 °C the disappearance of 10 mM hydrogen peroxide (cat. 21,676-3; Sigma-Aldrich), as described by Aebi^53^. One unit was defined as 1 µmol of H_2_O_2_ reduced/min. Five independent experiments were carried out.

### Measurement of total glutathione peroxidase (tGPX) activity

Total GPX (EC1.11.1.9) activity was assayed in a 50 mM KH_2_PO_4_ buffer (pH 7) supplemented with 1 mM EDTA, 1.5 mM NaN_3_, 1.3 mM reduced glutathione and 0.4 U/ml glutathione disulfide reductase (GR) (cat. S2002, cat. G4251, cat. G3664, respectively; Sigma-Aldrich). The enzyme assay mixture contained also 0.19 mM NADPH and 0.6 mM cumene hydroperoxide (cat. N7505 and cat. C0524, respectively; Sigma-Aldrich). The oxidation of NADPH was followed at 340 nm and 25 °C, according to Paglia and Valentine^54^. One unit was defined as 1 µmol of NADPH oxidized/min. Five independent experiments were carried out.

### Measurement of glutathione S-transferase (GST) activity

Total GST (EC 2.5.1.18) activity was recorded at 340 nm and 25 °C, by following the conjugation of 1 mM 1-chloro-2,4-dinitrobenzene (CDNB) with 2 mM reduced glutathione (cat. 13,863-0 and cat. G4251, respectively; Sigma-Aldrich), according to the method described by Habig and Jakoby^55^. One unit was defined as 1 μmol of GSH-conjugated product/min. Five independent experiments were carried out.

### Western immunoblot analysis

Control and ELF-MF-exposed cells were harvested and lysed (5 × 10^7^ cells/ml) in RIPA buffer (cat. R0278; Sigma-Aldrich) supplemented with protease inhibitor (cat. P8340; Sigma-Aldrich) and phosphatase inhibitors (cat. P2850 and P5726, both from Sigma-Aldrich). After centrifugation at 16,000 × g for 30 min at 4 °C, supernatants were assayed for total protein content, by using the BCA Protein Assay Kit and bovine serum albumin as the standard (cat. PR23225; Euroclone). Denatured samples (10–20 µg) were run in triplicates on polyacrylamide gels (9–14%), according to Laemmli^56^. Protein bands were transferred onto polyvinylidene difluoride (PVDF) sheets by electrophoretic transfer^57^. Non-specific binding sites were blocked at room temperature for 1 hour with 5% (w/v) Blotting-Grade Blocker (cat. 170–6404; Bio-Rad Laboratories s.r.l., Milan, Italy), in Tris-buffer saline containing 0.05% (v/v) Tween-20 (cat. P5927; Sigma-Aldrich) (TBS-T). Membranes were incubated overnight with the primary antibodies diluted in TBS-T, and then with the peroxidase-conjugated secondary antibody for 2 h (see the 2.1 Antibodies section for informations about dilutions used in this work). The specific immune complexes were detected and analyzed by using either Enhanced Chemiluminescent Substrate Kit (cat. EMP001005; EuroClone S.p.A., Milan, Italy) and Alliance LD2 hardware and software (UVItec Limited, Cambridge, UK) or Metal Enhanced DAB Substrate Kit (cat. 34065; Thermo Fisher Scientific, Inc.) and TotalLab (TotalLab Ltd, Newcastle upon Tyne, UK). β-actin was used as the loading control for data normalization. Results were given as arbitrary units. Four independent experiments were carried out.

### Immunofluorescence analysis

Cells were harvested and seeded (7.5 × 10^3^ cells/cm^2^) and grown on poly-L-lysine (cat. P-1399; Sigma-Aldrich)-coated coverlips. Control and ELF-MF-exposed cells were washed twice with DPBS without Ca^2+^/Mg^2+^ (cat. BE17–512F; Euroclone), fixed in 4% (w/v) paraformaldehyde in phosphate buffered saline (PBS) for 10 min, and permeabilized in PBS containing 0.05% (v/v) Triton X-100 for 10 min at 4 °C. Non-specific binding sites were blocked with 3% (w/v) BSA in phosphate buffered saline containing 0.1% (v/v) Tween-20 (cat. P5927; Sigma–Aldrich) (PBS-T) for 30 min. Then, cells were incubated with primary antibodies that were diluted in PBS-T containing 1% (w/v) BSA at 4 °C overnight (see the 2.1 Antibodies section for informations about dilutions used in this work). After extensive washes with PBS-T and a second incubation with 1% (w/v) BSA in PBS-T for 15 min, cells were incubated with AlexaFluor 488-containing PBS-T, in presence of 1% (w/v) BSA at 4 °C for 1 hour (see the 2.1 Antibodies section for informations about dilutions used in this work). After three washes with PBS-T, cells were mounted with Vectashield mounting medium containing (4′,6-diamidino-2-phenylindole) (DAPI) (cat. H-1200; Vector Laboratories). Cells were observed and photographed by fluorescence microscopy by using an Axio Imager A2 with Leica camera DFC320 (Carl Zeiss S.p.A., Milan, Italy). Digital images (12 fields/condition, three replicates; range of cells analyzed: 181–351) were analyzed by open source Java-based Fiji-ImageJ image processing package^58^, as recommended by the manufacturer. Results were given as mean gray values.

### Protein carbonyl content assessment

In order to detect the carbonyl content in our samples, a 2,4-dinitrophenylhydrazine (DNPH)-based Protein Carbonyl Colorimetric Assay Kit (cat. 10005020; Cayman Chemical Company, Ann Arbor, MI, USA) was used^59^. Briefly, control and ELF-MF-exposed cells were harvested and homogenized in cold EDTA-containing phosphate buffer (pH 6.7) (2 × 10^7^ cells/ml). Samples were centrifuged at 16,000 × g for 30 minutes at 4 °C. Supernatants were treated with 1% (w/v) streptomycin sulfate (cat. sc-202821; Santa Cruz Biotechnology, Inc., Santa Cruz, CA, USA) to remove contaminant nucleic acids, as recommended by the manufacturer. After the reaction with DNPH, samples were transferred into 96-well plate, and the formation of the corresponding hydrazones was recorded at 370 nm (extinction coefficient: 0.022 µM^−1^ × cm^−1^) by using a Victor3 microplate reader (PerkinElmer Inc.). As the several washing steps may easily cause protein loss, protein levels were determined on the post-washing final pellets, as recommended by the manufacturer. Five independent experiments were carried out.

### Alkaline single cell gel electrophoresis (SCGE)

In order to assess the basal level of oxidative damage to DNA, an alkaline single cell gel electrophoresis (SCGE) was carried out^60, 61^. Briefly, control and ELF-MF-exposed cells were harvested and re-suspended in 0.7% (w/v) low melting point (LMP) agarose (1.8 × 10^5^ cells/ml), applied onto microscope slides coated with a layer of 1% (w/v) agarose dissolved in PBS, and maintained at 4 °C for 5 min. A second layer of 0.7% (w/v) LMP agarose was pipetted onto microscope slides. Triplicate slides were prepared for each sample. Slides were submerged for 1 hour in a solution containing 2.5 M NaCl, 100 mM EDTA, 10 mM Tris, pH 10, 1% (v/v) Triton X-100 and 10% (v/v) dimethylsulfoxide (DMSO) (all from Sigma-Aldrich), at 4 °C. DNA in lysed cells was allowed to unwind in alkaline buffer (300 mM NaOH, 1 mM EDTA) for 20 min. Samples underwent electrophoresis for 30 min at a voltage of 1 V × cm^−1^. After electrophoresis, slides were neutralized in 0.4 M Tris-HCl pH 7.5, incubated in cold ethanol for 10 min, and left to dry overnight. Slides were stained by using ethidium bromide (20 μg/ml) for 5 min, observed under an Axio Imager A2 with Leica camera DFC320 (Carl Zeiss S.p.A.), and the resulting images (15 fields/condition; range of cells analyzed: 443–593; on average, ~30 cells/field) were processed by using CASPLab v1.2.3beta2 software to score comets, as described by Olive^62^.

### Evaluation of H_2_O_2_-dependent cytotoxicity

Control and ELF-MF-exposed cells were treated for 2 hours with either 35 µM H_2_O_2_ (cat. 21,676-3; Sigma-Aldrich) or sterile _dd_H_2_O dissolved in 1% (v/v) FBS-supplemented RPMI-1640 medium, containing 2mM L-glutamine. Low-serum medium was used to minimize both cell proliferation and protein-H_2_O_2_ interaction. The use of 35 µM hydrogen peroxide was decided on the basis of a 4P-logistic regression equation that was derived from a dose-response curve obtained by incubating control cells (i.e., non exposed) with different H_2_O_2_ concentrations (0, 25, 50, 100, and 200 µM). After the incubation with hydrogen peroxide, cells were detached and counted as described in the section 2.4 (i.e., viable cell counting). Results were given as % alive cells ﻿﻿﻿with respect to non treated cells. Four independent experiments were carried out.

### Evaluation of DOXO-dependent cytotoxicity

Control and ELF-MF-exposed cells were treated for 48 hours with either 90 nM doxorubicin (DOXO) (cat. D1515, Sigma-Aldrich) or sterile _dd_H_2_O dissolved in 1% (v/v) FBS-supplemented RPMI-1640 medium, containing 2 mM L-glutamine, 100 µg/mL streptomycin and 100 IU/mL penicillin. Low serum-containing medium was used to minimize cell proliferation. The concentration of doxorubicin (90 nM) was chosen on the basis of the reported continuous steady-state concentration achieved in blood during clinical treatments of children cancers^63, 64^. After the incubation, cells were detached and counted as described in the section 2.4 (i.e., viable cell counting). Results were given as % alive cells with respect to non treated cells. Five independent experiments were carried out.

### Statistics

Microsoft Excel 2007, Statsoft Statistica 10 and GraphPad Prism 6 packages were used for data processing, statistical analyses and visualization. Specifically, Mann-Whitney rank sum tests were used for non parametric datasets (i.e., SCGE- and immunofluorescence-related results, as well as all results from 100 µT MF exposure). Factorial ANOVA (2 × 2) and post-hoc Newman–Keuls/Tukey tests for multiple comparisons were used for all the remaining results, with the exception of those regarding H_2_O_2_- and DOXO-related cytotoxicity, that were analyzed by a (2 × 2 × 2) factorial ANOVA and Newman–Keuls/Tukey post-hoc tests. The null hypothesis was rejected with P < 0.05. With the exception of SCGE- and immunofluorescence-related results, that were reported as medians with interquartile ranges, all the other results were expressed as means ± standard deviations.

### Data availability

The datasets generated and/or analyzed during the current study are available from the corresponding author on reasonable request.

## Results

### Effects of ELF magnetic field exposure on cell growth

Our previous results^10, 12^ were entirely confirmed. In fact, a statistically significant increase in the number of alive neuroblastoma cells after exposure to 1 mT MF were detected (P < 0.001 vs time-matched non-exposed cells for both exposure times). In particular, after the exposure to the 1 mT MF the number of alive cells increased by 37.6% and 49.6% after 5 and 10 days, respectively, with respect to time-matched unexposed controls. As previously reported by some of us^10, 12^, no time-dependent effect was detected. These results suggested that the 50 Hz, 1 mT ELF-MF induced a powerful time-independent increase in biomass growth of neuroblastoma cell cultures. Surprisingly, SH-SY5Y cells that were exposed to the 1 mT MF continued to exhibit a statistically significant increase in the growth rate even after the power supply was switched off for 5 days (+43.8%, as compared to unexposed cells; P < 0.001), thus indicating that the hyperproliferative response of SH-SY5Y cells was not reverted by the removal of the MF. As a major endpoint, cell growth was also assessed after the exposure to a MF with much lower flux density (i.e., 100 µT). In cultures that were exposed to the 100 µT MF the number of alive cells increased by 23.4% and 14.9% after 5 and 10 days, respectively (P < 0.05 vs time-matched non-exposed cells for both exposure times), thus showing that the increase in biomass growth was still present with exposures to low-intensity MF.

### Effect of ELF-MF exposure on ROS-targeting enzymatic defence

**Figure 1:**
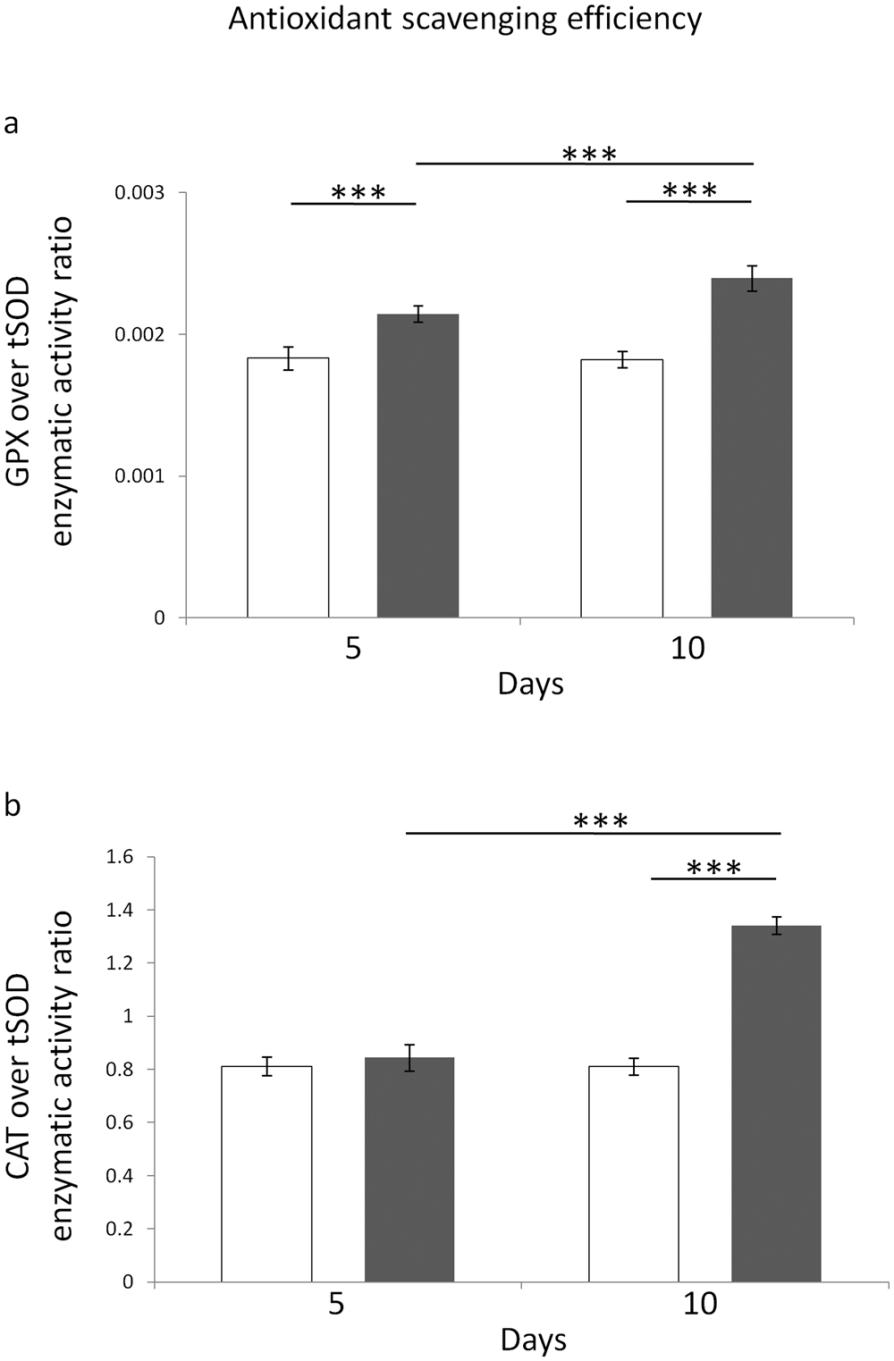
Effects of 5 or 10 days of ELF-MF exposure (1 mT, 50 Hz) on the major ROS-targeting antioxidant enzymatic defence systems in SH-SY5Y human neuroblastoma cells. Glutathione peroxidase (tGPX)/total superoxide dismutase (tSOD) (panel a) and catalase (CAT)/total superoxide dismutase (tSOD) (panel b) ratios were reported. Empty histograms represent unexposed cells (sham), whereas gray histograms represent ELF-MF-exposed cells. Values were expressed as means ± s.d. ***P < 0.001 (2 × 2 factorial ANOVA followed by post-hoc Newman–Keuls/Tukey tests for multiple comparison).

As already reported^65–67^, the ratio of specific activities of coupled antioxidant enzymes is much better indicative of the efficiency of the antioxidative enzymatic defence than each activity alone. Statistically significant main effects of both MF exposure and time on the GPX/tSOD ratio were found (F = 186.1, P < 0.001 and F = 13.86, P < 0.01, respectively). The post-hoc multiple comparison test revealed that the efficiency of GPX/tSOD antioxidative pathway was increased after both 5 and 10 days of ELF-MF treatment, with respect to the time-matched unexposed controls (P < 0.001 for both exposure times) (Fig. 1, panel a), however the MF-induced increase of GPX/tSOD ratio was greater after 10 days of treatment, with respect to that found after 5 days of exposure (P < 0.001) (Fig. 1, panel a), and this was confirmed by the significant statistical interaction between the two independent factors (F = 16.18, P < 0.01), which suggested that the effect of MF exposure on the GPX/tSOD balance was dependent on the exposure time.

Statistically significant main effects of both MF exposure and time on the CAT/tSOD ratio were also found (F = 278.3 and F = 215.6, respectively, P < 0.001 for both independent factors). The post-hoc test revealed that the efficiency of CAT/tSOD antioxidant pathway was increased only after 10 days of ELF-MF exposure, with respect to the time-matched unexposed controls (P < 0.001) (Fig. 1, panel b). Accordingly, a statistical difference (P < 0.001) was also found between 5- and 10-day ELF-MF-treated samples (Fig. 1, panel b), and this was confirmed by the significant statistical interaction between the two independent factors (F = 218.6, P < 0.001), which suggested that the effect of MF exposure on the CAT/tSOD balance was dependent on the exposure duration.

### Effect of ELF-MF exposure on macromolecular oxidative damage

**Figure 2:**
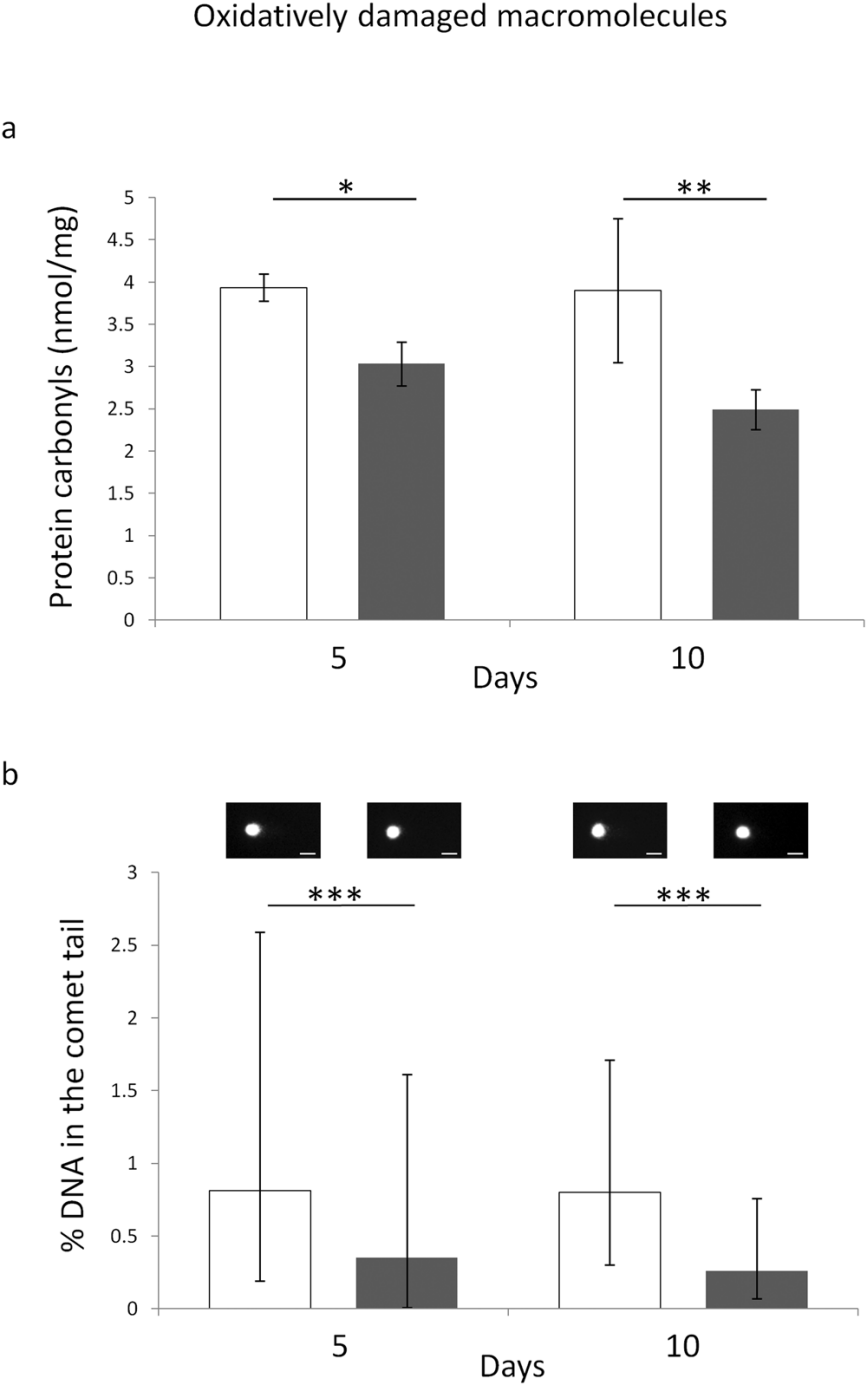
Effects of 5 or 10 days of ELF-MF exposure (1 mT, 50 Hz) on the levels of oxidatively modified proteins (panel a) and DNA (panel b) in SH-SY5Y human neuroblastoma cells. Empty histograms represent unexposed cells (sham), whereas gray histograms represent ELF-MF-exposed cells. Representative photographs of alkaline comets observed through fluorescence microscopy were reported in panel b (bar = 30 µm). Protein carbonyls were expressed as means ± s.d. Percentages of DNA in the comet tails were expressed as medians with interquartile ranges. *P < 0.05, **P < 0.01 (2 × 2 factorial ANOVA followed by post-hoc Newman–Keuls/Tukey tests for multiple comparison). ***P < 0.001 (Mann-Whitney rank sum tests).

A statistically significant main effect of MF exposure on the levels of protein carbonyls was detected by the two-way ANOVA (F = 30.63, P < 0.001). This was confirmed by the fact that the multiple comparison test revealed that the protein carbonyl content was decreased in a statistically significant fashion after both 5 and 10 days of ELF-MF exposure, with respect to the time-matched unexposed controls (P < 0.05 and P < 0.01, respectively) (Fig. 2, panel a).

Similarly, the level of oxidatively-modified DNA was decreased after both 5 and 10 days of ELF-MF exposure, with respect to the time-matched unexposed controls (P < 0.001 for both exposure times) (Fig. 2, panel b).

### Effect of ELF-MF exposure on SIRT1/3 and NRF2 protein levels

**Figure 3:**
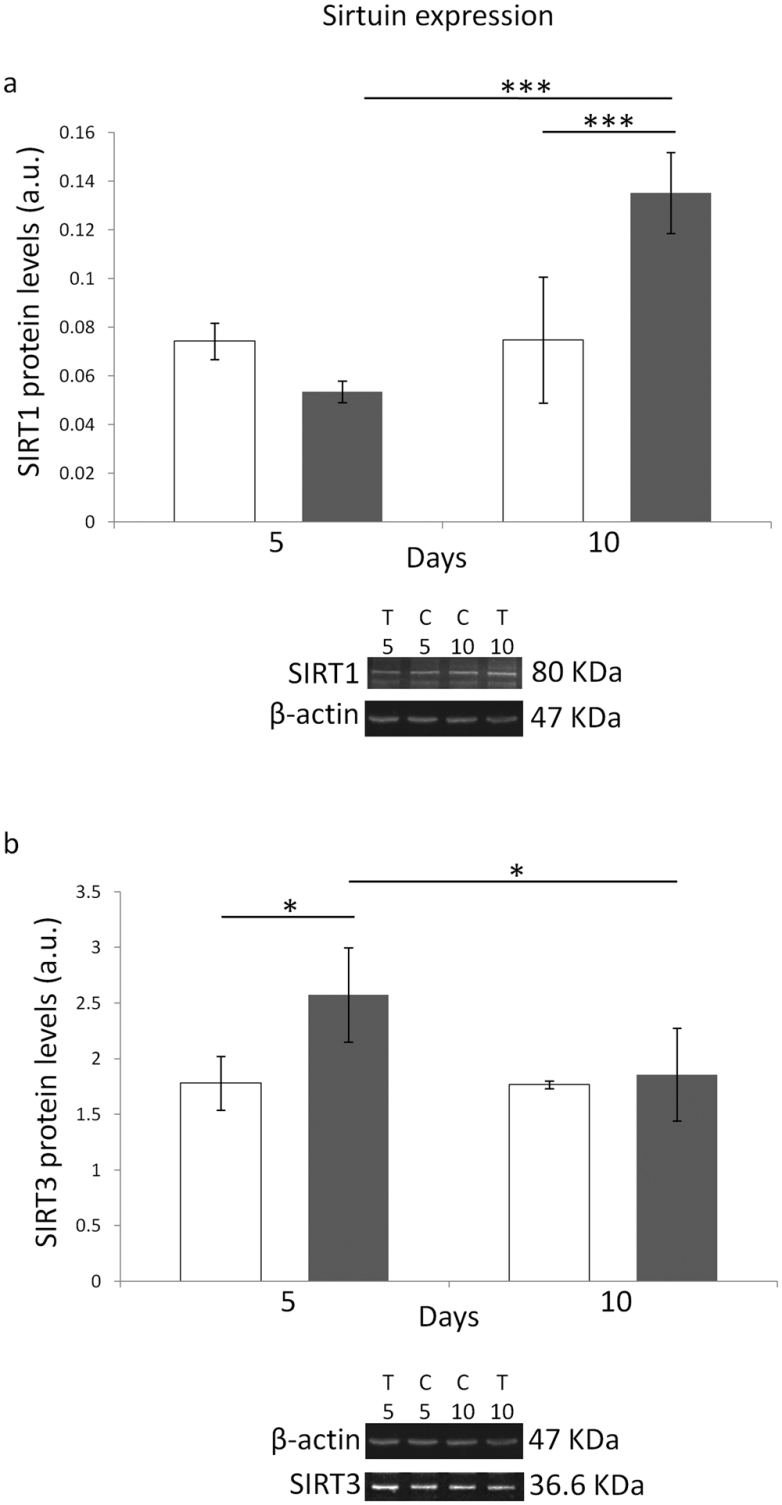
Effects of 5 or 10 days of ELF-MF exposure (1 mT, 50 Hz) on the protein levels of SIRT1 (panel a) and SIRT3 (panel b) in SH-SY5Y human neuroblastoma cells. Empty histograms represent unexposed cells (sham), whereas gray histograms represent ELF-MF-exposed cells. Representative grouping of Western blots were reported in both panels, with clear white space delineation between the proteins of interest (i.e., SIRT1 and 3) and the loading control (i.e., β-actin). T5 and T10, exposed cells for 5 and 10 days, respectively (C5 and C10, time-matched unexposed controls). Values were expressed as means ± s.d. *P < 0.05, ***P < 0.001 (2 × 2 factorial ANOVA followed by post-hoc Newman–Keuls/Tukey tests for multiple comparison).

Statistically significant main effects of both MF exposure and time on the SIRT1 protein level were detected (F = 6.131, P < 0.05 and F = 26.33, P < 0.001, respectively). The post-hoc test revealed that SIRT1 protein level was increased only after 10 days of ELF-MF treatment, with respect to the time-matched unexposed controls (P < 0.001), and a statistically significant increase (P < 0.001) was also found between 5- and 10-day ELF-MF-treated samples (Fig. 3, panel a). Such results were confirmed by the significant statistical interaction between the two independent factors (F = 25.63, P < 0.001), which indicated that the effect of MF exposure on the SIRT1 protein level was dependent on the exposure time.

With regard to the protein levels of SIRT3, statistically significant main effects of both MF exposure and time were detected (F = 7.617 and F = 5.203, respectively, P < 0.05 for both independent factors). The multiple comparison test showed that sirtuin 3 protein level was increased only after 5 days of ELF-MF treatment, as compared to the time-matched unexposed controls (P < 0.05) (Fig. 3, panel b). In addition, a statistically significant difference (P < 0.05) was also found between 5- and 10-day ELF-MF-treated samples (Fig. 3, panel b). The fact that the effect of MF exposure on the SIRT3 protein amount was dependent on the exposure duration was confirmed by the statistically significant interaction that was observed (F = 4.779, P < 0.05).

**Figure 4:**
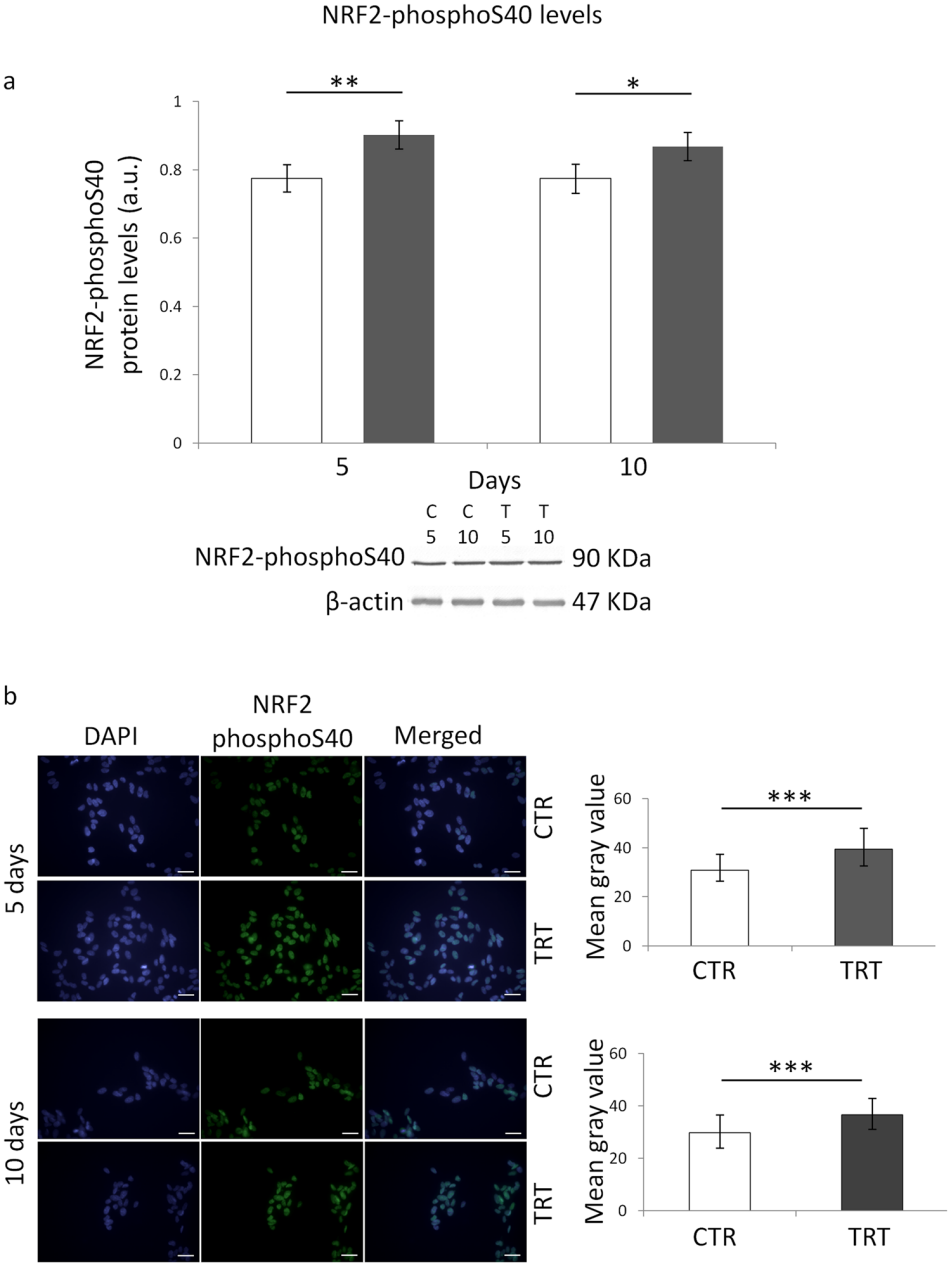
Effects of 5 or 10 days of ELF-MF exposure (1 mT, 50 Hz) on the protein levels of NRF2-phosphoS40 in SH-SY5Y human neuroblastoma cells. Empty histograms represent unexposed cells (sham, CTR), whereas gray histograms represent ELF-MF-exposed (TRT) cells. Representative grouping of Western blots were reported in panel a, with clear white space delineation between the protein of interest (i.e., NRF2-phosphoS40) and the loading control (i.e., β-actin). T5 and T10, exposed cells for 5 and 10 days, respectively (C5 and C10, time-matched unexposed controls). Representative photographs of immunofluorescence microscopy were reported in panel b (bar = 40 µm). Western blotting-related results were expressed as means ± s.d. Immunofluorescence-related results were expressed as medians with interquartile ranges. *P < 0.05, **P < 0.001 (2 × 2 factorial ANOVA followed by post-hoc Newman–Keuls/Tukey tests for multiple comparison). ***P < 0.001 (Mann-Whitney rank sum tests).

A statistically significant main effect of MF exposure on NRF2-phosphoS40 (i.e., the active form of NRF2) protein level was found (F = 28.68, P < 0.001). The multiple comparison test showed that the protein level of phospho-NRF2 was increased after both 5 and 10 days of exposure to the ELF field, with respect to time-matched unexposed controls (P < 0.01 and P < 0.05, respectively) (Fig. 4, panel a). The immunofluorescence microscopy-based investigation fully confirmed the Western blot data. In fact, the measurement of immunoreactivity revealed that NRF2 nuclear levels were increased by both 5- and 10-day ELF-MF exposure (P < 0.05 for both exposure times) (Fig. 4, panel b).

### Effect of ELF-MF exposure on H_2_O_2_-dependent cytotoxicity

**Figure 5:**
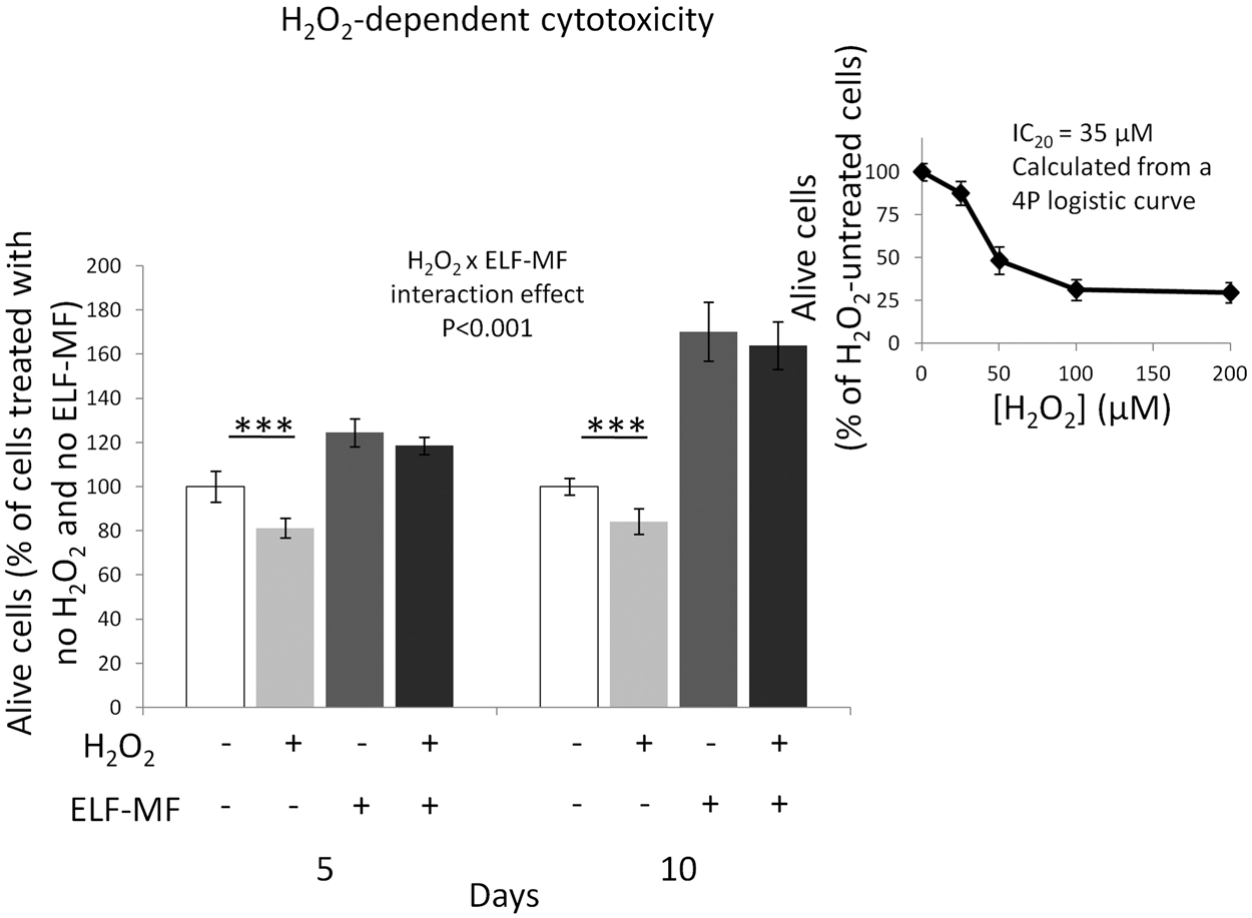
Effects of 5 or 10 days of ELF-MF exposure (1 mT, 50 Hz) on the survival of SH-SY5Y neuroblastoma cells after a 2-h incubation with 35 μM H_2_O_2_. Values were expressed as means ± s.d. A 20% decrease of alive cells (IC_20_) was calculated through a 4P-logistic regression derived from a dose-response curve obtained by incubating unexposed cells with H_2_O_2_ concentrations ranging from 0 to 200 µM (inset diagram). ***P < 0.001 (2 × 2 × 2 factorial ANOVA followed by post-hoc Newman–Keuls/Tukey tests for multiple comparison).

As expected, statistically significant main effects of both ELF-MF exposure and H_2_O_2_ treatment on alive cell number were detected (F = 1,183.46 and F = 58.07, respectively, P < 0.001 for both independent factors), and a statistically significant interaction between H_2_O_2_ treatment and ELF-MF exposure was also found (F = 13.01, P < 0.001), thus meaning that the H_2_O_2_-induced inhibition of growth was dependent on the MF-related status. Accordingly, the post-hoc test revealed that the H_2_O_2_-dependent reduction of alive cell number was statistically significant only in unexposed cells (P < 0.001 for both exposure times), whereas in ELF-MF-exposed samples the 2-hour H_2_O_2_ incubation did not reduce the number of alive cells (Fig. 5), at least from a statistically point of view. No statistical interaction among H_2_O_2_ treatment, ELF-MF exposure and time was found, thus meaning that the exposure duration did not affect the cellular response to H_2_O_2_, neither in MF-treated cells, nor in sham-controls.

### Effect of ELF-MF exposure on DOXO-dependent cytotoxicity

**Figure 6:**
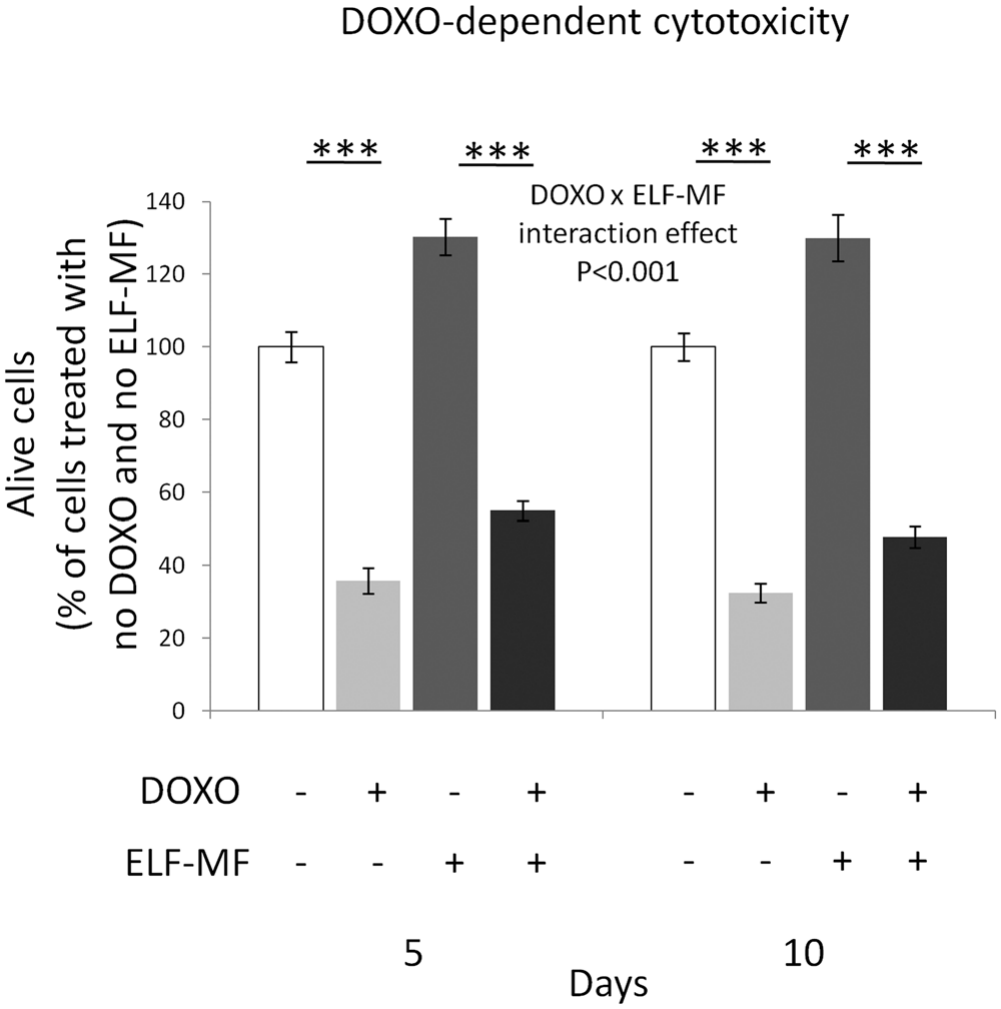
Effects of 5 or 10 days of ELF-MF exposure (1 mT, 50 Hz) on the survival of SH-SY5Y neuroblastoma cells after a 48-h incubation with 90 nM doxorubicin (DOXO). The concentration of doxorubicin was chosen on the basis of the reported continuous steady-state concentration achieved in blood during clinical treatments of children cancers^63, 64^. Values were expressed as means ± s.d. ***P < 0.001 (2 × 2 × 2 factorial ANOVA followed by post-hoc Newman–Keuls/Tukey tests for multiple comparison). For data derived from the 100 µT ELF-MF exposure, please refer to the 3.6 Results section.

As expected, main effects of both MF exposure and DOXO treatment on alive cell number resulted to be statistically significant (F = 304.41 and F = 2,759.9, respectively, P < 0.001 for both independent factors). The post-hoc multiple comparison revealed that the DOXO-dependent decrease of alive cell number was statistically significant both in unexposed and MF-exposed cells (P < 0.001 for both conditions, after both exposure times), and this indicated that the cytotoxicity of doxorubicin was not completely abolished by the pre-conditioning of cells with the ELF-MF. However, the DOXO-induced reduction of alive cells was less in ELF-MF-exposed samples than in unexposed cultures (Fig. 6). In particular, the percentages of post-DOXO alive cells were greater in cultures that were exposed to the MF both after 5 and 10 days of treatment, with respect to unexposed time-matched controls (+6.46% and +4.36%, respectively), and this was confirmed by the statistically significant interaction between DOXO treatment and MF exposure (F = 20.855, P < 0.001), which suggested that the effect of MF exposure on the DOXO-induced cytotoxic response was dependent on the exposure duration. These results were further confirmed by the dose-response curves that were obtained by treating with doxorubicin both MF-exposed and control cells (concentrations ranging from 0 to 400 nM), which revealed that the MF exposure increased the DOXO IC_50_ by 14.2%, specifically from 67.6 nM to 77.2 nM. Interestingly, MF-exposed SH-SY5Y cells continued to exhibit an increased resistance to 90 nM DOXO (+6.6%, as compared to unexposed cells; P < 0.05), even after the power supply was switched off for 5 days, thus suggesting that the MF-induced increase in cellular resistance against DOXO cytotoxicity was not reverted by the removal of the physical agent. As a major endpoint, DOXO-dependent cytotoxicity was also assessed after cells were exposed to a MF with much lower flux density (i.e., 100 µT). The percentages of post-DOXO alive cells were still greater in cultures that were exposed to the 100 µT MF, both after 5 and 10 days of treatment, in comparison with unexposed time-matched controls (+6.40% and +4.24%, respectively; P < 0.05 for both exposure times;), thus showing that the increase in cell resistance against DOXO was still present with exposures to low-intensity MF.

### Effect of ELF-MF exposure on GST-related detoxification activity

**Figure 7:**
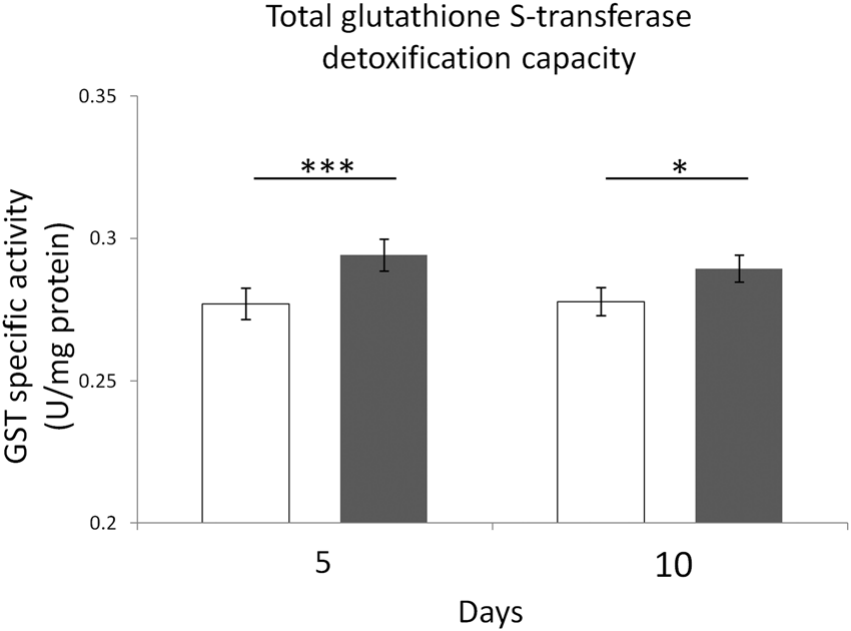
Effects of 5 or 10 days of ELF-MF exposure (1 mT, 50 Hz) on one of the major detoxification enzymatic defence systems in SH-SY5Y human neuroblastoma cells. The specific activity of glutathione S-transferase (GST) was reported. Empty histograms represent unexposed cells (sham), whereas gray histograms represent ELF-MF-exposed cells. Values were expressed as means ± s.d. *P < 0.05, ***P < 0.001 (2 × 2 factorial ANOVA followed by post-hoc Newman–Keuls/Tukey tests for multiple comparison).

A statistically significant main effect of MF exposure on the GST specific activity was found (F = 38.18, P < 0.001). The post-hoc multiple comparison test revealed that the increase of the specific activity of GST was statistically significant after both 5 and 10 days of ELF-MF exposure, with respect to the time-matched unexposed controls (P < 0.001 and P < 0.05, respectively) (Fig. 7).

## Discussion

Our study shows that a 50 Hz, 1 mT ELF magnetic field affects key aspects of human neuroblastoma cell behaviors that are critically linked to malignant phenotype, such as cell growth, and cellular resistance towards clinically relevant ROS-generating treatments. Such cellular responses are coupled with important improvements in the activity of the major antioxidant and detoxification defensive systems, as well as by the activation of crucial regulators of the cellular redox environment.

Our experiments revealed that the marked ELF-MF-induced increase in cytoproliferation of neuroblastoma cells was accompanied by a statistically significant improvement of key ROS-scavenging capacities (Fig. 1), which in turn was associated with diminished levels of oxidatively-modified biomolecules (i.e., proteins and DNA) (Fig. 2). In coherence with the observed activation of the main antioxidant enzymatic defences, three of the major redox-responsive controllers of cellular response towards oxidative challenge (namely, SIRT1/3 and NRF2) were found to be strongly upregulated by the treatment with the ELF field (Figs 3 and 4). ELF-MF-induced hyperproliferation of NB69 human neuroblastoma cells has already been reported to be linked to the activation of ROS-responsive pathways (i.e., extracellular signal-regulated kinases, ERK)^68^, that are known to be strictly involved in the upregulation of NRF2^69^. Accordingly, our experiments brought to light that nuclear NRF2 was increased in cells that were exposed to the ELF field (Fig. 4) in a statistically significant fashion. A 12-day exposure to a 50 Hz, 1 mT ELF-MF has been reported to increase SIRT1 level in mesenchymal stem cells from adult bone marrow^46^, whereas transcranial magnetic stimulation with an ELF field has been proven to upregulate NRF2 in a rat model of Huntington’s disease^45^. Since SIRT1 and SIRT3 overexpression has been previously shown to elicit cytoprotective action in SH-SY5Y against oxidative stress-promoting challenges^70, 71^, the distinctive pattern of biochemical and molecular responses we observed in MF-exposed cells corroborate the idea that the interaction between ELF-MF and biosystems may activate redox-responsive processes, as hypothesized by us and others^19, 20, 25, 26, 72, 73^.

The activation of antioxidative mechanisms is usually considered as a feature that reflects a healthy cellular status. Therefore, at first glance, our findings could be considered as an indication of a positive effect of the ELF magnetic field. On the other hand, it must be taken into account that a particularly healthy hyperproliferating malignant cell may acquire critical survival advantage. In fact, an elevation of the ROS-targeting activity is currently believed to promote tumor progression and multidrug resistance^14, 15^. Accordingly, gene expression array data show that key antioxidant enzymes are strongly overexpressed in metastatic cells, when compared to primary tumors^74^. Moreover, many oncogenes are known to code for redox-sensitive transcription factors that are able to induce a complex set of adaptive responses, providing a survival advantage under chronic redox stress, and contributing to the development of chemoresistance^18, 75^. For these reasons, most of antitumor agents kill cancer cells by overwhelming their high ROS scavenging capacity and inducing oxidative stress^16, 76^. Here we report that ELF-MF is also able to activate important ROS-targeting cytoprotective mechanisms that may be possibly linked to tumor progression and chemo-resistance. Some of us have already reported that ELF-MF enhances the efficiency of enzymatic systems that target endogenously overproduced cancer-static dicarbonyl metabolites in cancer cells, thus creating the basis of multidrug resistance in neuroblastoma cells^12^. In order to verify whether the ELF-MF-induced improvement of antioxidant defensive systems may be associated with higher resistance of neuroblastoma cells towards ROS-generating treatments, we investigated whether the vulnerability of neuroblastoma cells towards both hydrogen peroxide and the anti-tumor drug doxorubicin (DOXO) could be affected by the ELF-MF exposure. Interestingly, we found that the ELF field exposure abolished the toxic effect of hydrogen peroxide on SH-SY5Y cells (Fig. 5), whereas the loss of alive cells after DOXO incubation was weakened in ELF-MF-exposed cultures (Fig. 6) in a statistically significant fashion. Even though the effect of the ELF-MF on DOXO-induced cytotoxicity may appear small, it should be remembered that tumor and cancer growth is strongly dependent on both cell proliferation and survival. Noteworthy, uncontrolled clonal doubling in malignancies is undoubtedly an exponential process, and this means that even small differences in proliferative kinetics may have relevant effects on dynamics of tumor growth and final cell numbers, especially in our case in which the doubling time of MF-treated cells decreases by more than 10%^10, 12^. In coherence with our findings, NRF2 upregulation has been recently linked to the acquisition of resistance against doxorubicin and other anticancer drugs in several cancer cell types^44, 77, 78^. Moreover, the doxorubicin-induced toxicity in rat neonatal cardiomyocytes was shown to be prevented by SIRT3 activators^79^. Hence, the chemical suppression of both SIRT3- and NRF2-dependent signaling pathways is currently seen as an effective means by which cancer cells could be sensitized to chemo- and radio-therapy^37, 39, 80^. The shift of cancer metabolism from glycolysis to mitochondrial oxidative phosphorylation is associated with the development of resistance to chemotherapy, and this process seems to be promoted by the enhancement of SIRT1/PCG1α-driven ROS scavenging defences^40^. In our previous work we demonstrated that the ELF-MF exposure increased the protein level of the PGC-1α in SH-SY5Y cells. Now we extend such biomolecular landscape by showing that ELF-MF treatment may be able to activate the PCG1α-dependent pathway by increasing SIRT1 expression, thus improving the cellular capacity to respond to pro-oxidant challenges. Last but not least, our investigation also revealed that the MF increased the specific activity of glutathione S-transferase (Fig. 7), which is one of the most important NRF2-controlled phase II enzymes that is frequently responsible for chemoresistance in cancers through the conjugation of xenobiotics to glutathione^31^. In coherence with the crucial role of GST in cancer drug resistance, the pharmacological suppression of GST activity has been recently shown to reverse the resistance of small-cell lung cancer cell line to vincristine and doxorubicin, two compounds that are commonly used in cancer chemotherapy^81^.

In brief, our results indicate that the several MF-induced adaptations may provide a mechanism of resistance to anticancer treatments that acts on a redox basis. The aggressiveness of malignancies is known to correlate to the degree of antioxidant capacity^28, 48^, and this highlights the importance of our findings.

Indoor environments, where multiple domestic appliances are often used simultaneously, are frequently associated with ELF-MF within the mT range^49^, however important biological alterations are strongly suspected to derive from *in vitro* exposure to ELF magnetic fields considerably lower than 1 mT^82^. For this reason, we wanted to verify whether the major effects that were elicited in neuroblastoma cells by a 1 mT MF were also detected when cells were exposed to a 50 Hz, 100 µT MF, which is 10-fold below the new reference level established in 2010 by the International Commission on Non-Ionizing Radiation Protection guidelines for occupational exposures to power frequency ELF magnetic fields^49^). Interestingly, our experiments showed that both MF flux densities caused an increase in the biomass growth rate, as well as the development of similar degree of drug-resistance against doxorubicin in SH-SY5Y cells, and this, in turn, may suggest that the response of human neuroblastoma cells to the MF is not strictly dependent on the magnitude of the MF used. In other words, our experimental results suggests that exposure to 50 Hz magnetic fields as low as 100 µT may still be able to alter behavior and responses of cancer cells to clinically-relevant drugs.

Lastly, some authors have suggested that the cellular effects that are induced by ELF-MF should be considered as transient^83^, however, we here demonstrate that not only were both cellular behavior and biomolecular profile of neuroblastoma cells profoundly modified by the exposure to the 1 mT ELF field, but also such major clinically relevant bioeffects were not reversible, at least within the time range considered in our follow-up experiment. In fact, MF-exposed cells continued to exhibit both increased growth rate and enhanced resistance to DOXO-related cytotoxicity, as compared to unexposed cells, even after the power supply was switched off for 5 days. This observation represents a particularly important finding as it implies that the perturbation of cancer cell behavior may derive from a biomolecular rewiring that persists after the initial interaction between MF and the cellular system. Such phenomenon might suggest that permanent ELF-MF-induced changes in the transcriptional and/or translational profile of exposed cells may occur.

In conclusion, our study reports that the biology of neuro-derived malignant cells is altered by ELF-MF exposure. This could provide proliferative or survival advantage through an enduring activation of major redox-responsive antioxidative and detoxification cytoprotective pathways that are associated with more aggressive/resistant behavior.

To the best of our knowledge, we are among the firsts to demonstrate that some of the major redox-related pro-malignant effects of a 50 Hz, 1 mT ELF-MF are associated with the development of clear traits of drug-resistance, and that such alterations are not immediately reverted by the removal of the magnetic field. Moreover, the existing literature on this research topic is predominantly based on short exposure durations, and not on chronic exposure paradigms.

It has to be pointed out that *in vitro* data presented here should not be automatically extended to *in vivo* situations, and that more studies in this field are certainly needed. However, we think that the molecular targets uncovered in this work, if appropriately confirmed *in vivo*, might help to develop innovative approaches aimed at limiting the deleterious effects of ELF magnetic fields in malignant cells.
